# Supplementary material for: Comparison of stochastic and deterministic models for gambiense sleeping sickness at different spatial scales: A health area analysis in the DRC
Source: PLoS Comput Biol. 2024 Apr 1;20(4):e1011993. doi: 10.1371/journal.pcbi.1011993 (PMC11008881; doi:10.1371/journal.pcbi.1011993)
Supplement: Attachment — Submitted filename: Reviewer_response.pdf [file pcbi.1011993.s003.pdf]

Dear Alex Perkins and Thomas Leitner,

Thank you for these reviews of our manuscript, which have highlighted some important ways that it could be improved. We believe we have addressed these concerns in an updated version (see our track changes version) and have included a detailed table containing all our responses to reviewer comments.

Best wishes,
Christopher Davis
On behalf of all co-authors

Reviewer #1

Comment	Response
A scheme should be though out for identifying sub-figures (use A, B, C, D for example). Right now it is not obvious by reading the caption which sub-figure is being referred to.	We have added lettered labels to all multi-panel figures and referred to these letters in figure captions and within text references where appropriate.
In the methods section, give the equation between lines 246 and 247 a number. It seems like that equation number is out of the page. The second comment is regarding the description of terms in this equation. Define Bin(m;n,p) as well along the same footing as BetBin(m;n,p,rho). Can you also explain the reason for each denominator in the likelihood equation in the text just as you explain z(i)?	We have labelled the equation and added a definition for Bin(m;n,p). We have also added an additional paragraph to describe all the probability terms p.
In the discussion section, line number 426, the word using comes twice at the beginning of this line.	Corrected the typo.
Line 505 . the word 'no' should be 'not'.	Corrected the typo.
In general please run a spell check and also look for repeating words etc.	Thank you for noting this. A more rigorous spell-checker corrected a few small errors.
It would be beneficial if the authors could demonstrate the robustness of their findings by varying disease parameters. The use of toy data to examine the point at which calibration on deterministic and stochastic models diverge in small community sizes could help strengthen their argument.	In the SI, we have added toy data projections for small population sizes of N_H=10,000 and 1,000 to show that the expected outputs of the models do not diverge even in these cases. And we note that for N_H=100, there are zero expected cases and PEoT very close to one in both deterministic and stochastic models. We have also shown the PEoT for these three population sizes alongside the real data, which have an earlier PEoT projected for smaller population sizes, as stochastic

extinction is more likely. As with the real data, in all cases there is still more uncertainty in inferred new infections and therefore predicted elimination times in the stochastic model.

We have also considered varying the underlying fixed disease parameters. Most of these were chosen to be fixed as they are reasonable estimations for them in the literature and thought to be geographically invariant, but since our belief is that the similarities in deterministic and stochastic outcomes for gHAT are linked to the long time span of infection, we explored if changing this lead to more stochastic extinctions. For this exercise, the infection period was reduced by a factor of 10 and the effective tsetse density increased by a factor of 10 (to maintain the same R_0 value, a fitted parameter), we see a much earlier expected time for elimination. We do not include these results in the manuscript but demonstrate these results below. The long time scale of HAT infection are indeed key to the dynamics and the slow disease progression makes elimination a more difficult goal.

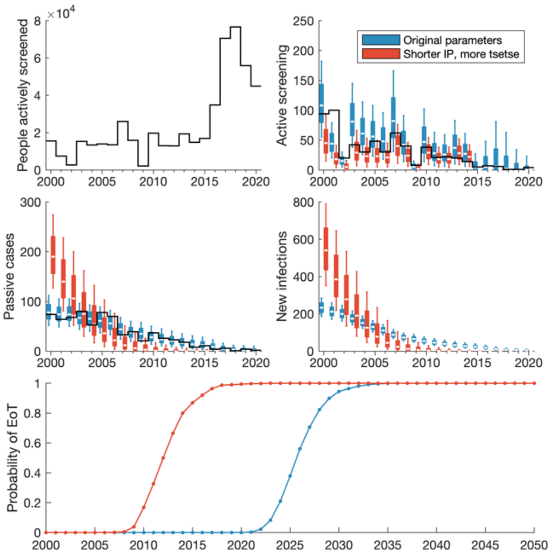

Reviewer #2

Comment	Response
Include a fit of the model to a health zone other than the primary one used in model development. This would demonstrate the flexibility of the model, and comparison of the fitting process in two different contexts could lead to additional insights into how to best use this model. Implementation in a zone where tsetse control was used could be particularly illuminating.	We have added a health zone fit for a neighbouring health zone, Yasa Bonga (in which tsetse control has been implemented since 2015) into the SI, as suggested. This is now referred to from the main text.<br><br>Previous work demonstrates this model is flexible. Health zone and focus fits have been carried out in several previous publications (Crump 2021, Rock 2022, Kaba 2023 etc), including where tsetse control has been implemented, across the DRC as well as foci in Chad and Cote d'Ivoire. We have now emphasised this within the methods section.
The Open Science repository of code should be mentioned in the main text of the paper, and the information and structure of the repository should be improved to facilitate the use of this code by other researchers. Some ideas for how to do this are available from these workshop notes: https://epiverse-trace.github.io/research-compendium/beforestart.html	We mention at the beginning of the Materials and methods section where the model code can be found. We have made this clearer by stating the link is to the Open Science Framework.<br><br>We have also restructured within the zip files to make the code more understandable, added a README file into the repository which includes installation and usage, and added an example of the data format required to run the model (but not the data itself which must be requested from the WHO to gain access).<br><br>We strongly support the work of the epiverse project to standardise code used for epidemiological research, however our project significantly predates the publication of this guidance and retrospectively adjusting our work to fit its framework would require a very substantial quantity of work.
As a smaller issue, I checked some of the sources in table 1 and found it hard to figure out the justification for some of the parameters. For instance, the active screening sensitivity has a citation to a paper that calculates sensitivity for specific	Thank you for checking our fixed parameter values against their sources — this is useful for ensure we are using correct values. However, we do believe that the parameter values are accurate.

regions not including DRC. This seems to contradict line 191 saying that sensitivity is "fitted to match data in the study area". The authors seem to be using the estimate for the Republic of Congo, some explanation of why this is justified would be appropriate. Another issue is that the given mortality rate gives a life expectancy of 50 years, which seems to contradict the cited source which gives the life expectancy as 59. Why this discrepancy?<br>If the above two issues with Table 1 turn out to have some substance then it would be good to double-check all the parameter sources.	For sensitivity we have used a value averaged from the citation to get a good estimate. This is not meant to contradict line 191, where this only applies to the specificity — we fit specificity but used a fixed sensitivity value. We have clarified the text to avoid this confusion: "We assume the diagnostic algorithm has a sensitivity of 91% (using a value averaged from Checchi et al.), while the specificity has been fitted to match data in the study area." We also note that we would not expect a significant geographical variation in the sensitivity values of individual tests, so think the use of data from the Republic of Congo is appropriate with a lack of data from the DRC. In particular, DRC and Republic of Congo used the same diagnostic algorithm and are geographic neighbours so we feel it is reasonable to assume similar sensitivity values.<br><br>For the life expectancy, we use the value from the year 2000 (start of our simulations), which was correct at the time it was viewed (noted in the reference). It appears that this number has subsequently been updated from 50 to 52. Below is a screenshot of the historic webpage. The rate of natural mortality is also a very small rate compared to the disease progression/treatment and we would thus expect small changes to life expectancy to have minimal impact on our simulations.<br>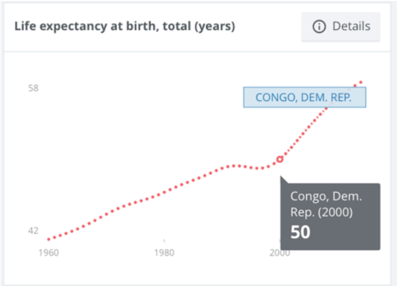
line 90: typo	Corrected the typo.
line 139-141: "supported anecdotally" is strange phrasing here, better to put this fact at the top of paragraph and use it as inspiration for the model structure	We have moved this information to the top of the paragraph on risk structure and removed the wording "supported anecdotally."

line 147: three different expose compartments not reflected in figure 2	We mechanically have three exposed compartments in the model to achieve our desired non-exponential distribution but choose not to present this explicitly in figure 2, since the sum of these three compartments reflect all people in our exposed class. We have added the words “Erlang distribution” on the E_V exit rate and have clarified our use of the linear chain trick for this compartment in the text.
line 190: typo	Corrected the typo.
line 400: I don't know what "comparable to the same calculation used" means here	We have removed the confusing wording and replaced with “comparable to the PEoT calculated for the health zone model.”
line 505: typo "no" vs "not"	Corrected the typo.
line 281: "rate of detection" is ambiguous here. Do you mean proportion of cases detected, or the rate at which new cases are reported?	We mean the rate at which new cases are reported and have amended the text.
