# Supplementary material for: Comparison of stochastic and deterministic models for gambiense sleeping sickness at different spatial scales: A health area analysis in the DRC
Source: PLoS Comput Biol. 2024 Apr 1;20(4):e1011993. doi: 10.1371/journal.pcbi.1011993 (PMC11008881; doi:10.1371/journal.pcbi.1011993)
Supplement: Attachment — Submitted filename: Reviewer_response2.pdf [file pcbi.1011993.s004.pdf]

Dear Alex Perkins and Thomas Leitner,

Thank you for these updated reviews of our manuscript. We have corrected the small error, changing the reference from S1 to S2.

Best wishes,
Christopher Davis
On behalf of all co-authors
